# Characterizing Heterogeneity within Head and Neck Lesions Using Cluster Analysis of Multi-Parametric MRI Data

**DOI:** 10.1371/journal.pone.0138545

**Published:** 2015-09-23

**Authors:** Marco Borri, Maria A. Schmidt, Ceri Powell, Dow-Mu Koh, Angela M. Riddell, Mike Partridge, Shreerang A. Bhide, Christopher M. Nutting, Kevin J. Harrington, Katie L. Newbold, Martin O. Leach

**Affiliations:** 1 CR-UK Cancer Imaging Centre, The Royal Marsden NHS Foundation Trust and The Institute of Cancer Research, London, United Kingdom; 2 Head and Neck Unit, The Royal Marsden NHS Foundation Trust, London, United Kingdom; 3 Radiology Department, The Royal Marsden NHS Foundation Trust, London, United Kingdom; 4 CRUK/MRC Oxford Institute for Radiation Oncology, University of Oxford, Oxford, United Kingdom; University of Pécs Medical School, HUNGARY

## Abstract

**Purpose:**

To describe a methodology, based on cluster analysis, to partition multi-parametric functional imaging data into groups (or clusters) of similar functional characteristics, with the aim of characterizing functional heterogeneity within head and neck tumour volumes. To evaluate the performance of the proposed approach on a set of longitudinal MRI data, analysing the evolution of the obtained sub-sets with treatment.

**Material and Methods:**

The cluster analysis workflow was applied to a combination of dynamic contrast-enhanced and diffusion-weighted imaging MRI data from a cohort of squamous cell carcinoma of the head and neck patients. Cumulative distributions of voxels, containing pre and post-treatment data and including both primary tumours and lymph nodes, were partitioned into k clusters (k = 2, 3 or 4). Principal component analysis and cluster validation were employed to investigate data composition and to independently determine the optimal number of clusters. The evolution of the resulting sub-regions with induction chemotherapy treatment was assessed relative to the number of clusters.

**Results:**

The clustering algorithm was able to separate clusters which significantly reduced in voxel number following induction chemotherapy from clusters with a non-significant reduction. Partitioning with the optimal number of clusters (k = 4), determined with cluster validation, produced the best separation between reducing and non-reducing clusters.

**Conclusion:**

The proposed methodology was able to identify tumour sub-regions with distinct functional properties, independently separating clusters which were affected differently by treatment. This work demonstrates that unsupervised cluster analysis, with no prior knowledge of the data, can be employed to provide a multi-parametric characterization of functional heterogeneity within tumour volumes.

## Introduction

Magnetic resonance imaging (MRI) is a highly flexible functional imaging (FI) technique enabling evaluation of multiple aspects of tumour biology in a single examination without radiation exposure [1,2], in contrast to modalities such as positron emission tomography (PET) or contrast-enhanced computer tomography (CT) [1,3]. Dynamic contrast-enhanced (DCE)-MRI provides information on tumour microcirculation, vascularity, blood volume and vessel permeability [4]; diffusion-weighted imaging (DWI) measures differences in tissue microstructure related to tumour cellularity, tissue disorganization, and increased extracellular space tortuosity [5]; intrinsic susceptibility imaging (IS) provides information relating to the oxygenation of tissues [6]. Assessment of changes in FI parameters during treatment could lead to a better understanding of the mechanism of treatment resistance or sensitivity [3]. A combination of FI parameters, each contributing complementary information, is likely to offer increased specificity for differentiation of cancerous tissue [7].

There is a pressing need to reliably describe functional or biological heterogeneity within a tumour. This is particularly relevant in squamous cell carcinoma of the head and neck (SCCHN), a disease which has a worldwide incidence of approximately 500,000 cases per annum [8]. One of the main challenges in advancing the management of SCCHN is the inability to predict treatment outcome due to tumour heterogeneity [2]. Multi-parametric FI may identify potentially treatment resistant or sensitive tumour sub-volumes [3,9] and thereby influence treatment selection such as radiation dose boosts, use of hypoxic cell radiosensitisers [10,11], treatment de-escalation to reduce normal tissue toxicity [12,13], or a change of treatment modality.

When multi-parametric imaging is available, unsupervised clustering techniques can segment different tissue types using a combination of FI parameters [14]. These methodologies are frequently employed to extract anatomical features within the image or to separate the tumour from the surrounding tissue, and their reliability is often assessed against visual inspection. The possibility of applying similar clustering techniques to characterize functional heterogeneity within the tumour volume alone remains underexplored. In this situation, prior knowledge of the data or anatomical references are limited or absent, and therefore testing the partitioning for robustness and consistency is advisable prior to attempting an interpretation of the results. Specifically, the number of clusters, a parameter which is normally pre-set arbitrarily, requires optimization in order for the structures revealed in the data to be meaningful [15].

In this paper we present a methodology, based on cluster analysis, to partition multi-parametric FI data into groups (or clusters) of similar functional characteristics, combining information from both DCE and DWI parameters, with the aim of describing functional heterogeneity within tumour volumes. This approach employs cluster validation techniques and principal component analysis to both understand the structure of the data and to assess the consistency of the partitioning. This workflow was applied to a cohort of SCCHN patients receiving induction chemotherapy (IC) and undergoing MRI as part of a previously reported feasibility study [3]. The relationship between partitioned data and response to treatment was investigated in order to identify those parameters more likely to characterize treatment-resistant disease.

## Materials and Methods

### Clinical Examinations

As part of a prospective pilot study, described by Powell *et al* in [3], 9 SCCHN patients received two cycles of IC (cisplatin (75 mg/m^2^) day 1 and 5-FU (1000 mg/m^2^) days 1–4) followed by radical chemoradiotherapy (cisplatin (100 mg/m^2^) days 1 and 29); macroscopic and microscopic disease received 65 and 54 Gray (Gy) in 30 fractions, respectively, using intensity modulated radiotherapy (IMRT) with a simultaneous integrated boost technique). All patients underwent MRI at the following time-points: baseline, following IC, after 40 Gy of IMRT, three and six months post-treatment. This study has been approved by the UK National Research Ethics Service (EC 08/H0801/132), and written informed consent has been obtained from all the participants. This cohort included 6 stage IVA, 1 stage IVB and 3 stage III patients, with primary sites located as follows: 5 in the palatine tonsil, 2 in the pyriform fossa, 1 in the nasopharynx, and 1 in the oropharynx [3].

MRI was performed at 1.5T (Philips Intera, Philips Medical Systems, Best, Netherlands), with patients immobilized in a 5-point thermoplastic mask and lying on a flat rigid bed. Two flexible surface coils were centred over the tumour [16] and the following functional imaging sequences were acquired: DCE, following gadolinium injection (0.2 mg/kg, Dotarem®, Guerbet, France), with a transaxial 3D T1-weighted spoiled gradient-echo pulse sequence (TE = 1 ms, TR = 4 ms, FOV = 256x256 mm^2^, 2x2x5 mm^3^ voxel, 50 mm coverage in the superior/inferior direction, 1.5 s temporal resolution, with a parallel imaging factor of 1.7 in the anterior/posterior direction); DWI (multi-slice EPI, b = 0, 100, 500, 1000 s/mm^2^, TE = 91 ms, TR = 2000 ms, FOV = 256x256 mm^2^, 2x2x5 mm^3^ voxel, 20 slices, with SPAIR fat suppression). The DCE protocol acquired proton density-weighted images (FA = 4°), later used for signal conversion to contrast agent concentration, followed by 100 dynamic acquisitions (FA = 20°). DCE and DWI image volumes were aligned and had the same voxel size.

### Data Analysis

For each slice, regions of interest (ROI) were delineated on DCE data by a radiation oncologist together with an expert radiologist around all primary tumours (PT) and target lymph nodes (LN), employing the software package MRIW (Institute of Cancer Research, London, UK), which allows analysis of DCE-MRI data sets using standard pharmacokinetic models [17]. The outline of PTs and LNs was defined as the visible enhancing area immediately following carotid enhancement [18]. Once the onset of enhancement was identified for each voxel, the initial area under the gadolinium curve (IAUGC60—integration over 60 s from the onset) was computed inside the defined ROIs. Spatial registration of DWI and DCE images was performed using a rigid body mutual information algorithm [19] implemented in IDL (version 8.2, Exelis Visual Information Solutions, Boulder, Colorado, USA). After applying the resulting integer voxel shift to DWI data, apparent diffusion coefficient (ADC) maps were produced employing a mono-exponential model to fit all 4 b values, and the ROIs delineated on DCE data were transferred to the computed maps. For DWI data, an independent set of manually delineated ROIs was also produced, encompassing the area of impeded diffusion on the b = 1000 images.

### Cluster Analysis

#### Clustering algorithm

Cluster analysis was performed using in-house software coded in IDL. The K-means algorithm was employed to partition the ROIs into sub-regions of similar characteristics, defined in the two-dimensional feature space formed by the two parameters ADC and IAUGC60. The robust implementation of the K-means algorithm included in the software package CCHIPS [20] was incorporated into the code, as it provides reproducible results and is independent of starting conditions. The algorithm standardizes the data, accounting for the different scales of the parameters. Following cluster analysis, each voxel was assigned a colour, with different colours representing membership of a different cluster.

In order to obtain a generic classification of the data, applicable to the whole cohort, the clustering algorithm was applied to the cumulative distribution of voxels containing data from all the patients and including both baseline and post-IC data. The slice with the largest cross-section (referred to as “*central ROI*”) was selected for each lesion. The cumulative distribution containing pre and post-treatment *central ROIs* from both PTs and LNs was partitioned into k clusters (k = 2, 3 or 4).

After classification, the number of voxels assigned to different clusters within each lesion pre and post-treatment was counted. For each cluster, paired groups of voxel counts were compared using the Wilcoxon signed-ranks test; a two-tailed p-value < 0.05 was chosen to indicate a significant difference in the number of voxels between pre and post-treatment data.

In Appendix 1 pre and post-treatment voxels counts are reported in detail for the PT *central ROIs*, in the simplest case of k = 2, with the purpose of demonstrating how the proposed method analyses longitudinal data. Additionally, the PTs were re-analysed comparing the use of the *central ROIs* only versus the use of the entire PT volumes, in order to determine if the slice with largest tumour cross-section represents the tissue type content of the whole lesion.

#### Principal component analysis

In order to assess the relative contribution of each of the two parameters (IAUGC60 and ADC) to the partitioning, principal component analysis (PCA) [21] was performed on the cumulative distribution of voxels containing the *central ROIs* of both PTs and LNs.

#### Cluster validation

A fundamental problem in cluster analysis is the determination of the optimal number of clusters, i.e. the value of k which best describes the data [22]. The “jump method” [15] was employed to determine the value of k which maximizes efficiency while minimizing error by information theory standards. This method makes use of a measure of data dispersion within the cluster indicated as *transformed distortion* and defined as:

$$d_{k}=\frac{1}{p}\cdot \underset{c_{1}\ldots c_{k}}{\text{min}}\text{MAHAL}{\left(X,c_{x}\right)}^{-\frac{p}{2}}$$ (1)

Where k = number of clusters

p = number of dimensions of the feature space

**X** = p-dimensional data

**c**_**1**_…**c**_**k**_ = cluster centres, being **c**_**x**_ the one closest to **X**

MAHAL = Mahalanobis distance

This method was applied to the partitioned data and produced a *distortion* value for each k. The validation curve plots the difference between consecutive *distortion* values as a function of k, and was generated for the set of data containing the *central ROIs* of both PTs and LNs. The value of k associated with the largest “jump” in the validation curve represents the optimal number of clusters [15].

## Results

Fourteen separate lesions, 5 PTs and 9 LNs, were considered in the analysis of 9 patients. Although 25 lesions were depicted in this cohort, complete functional datasets were available only in 14 lesions, due to differences in superior/inferior coverage between the DCE and DWI sequences. A total of 2179 voxels from the *central ROIs* (1435 pre-treatment and 744 post-IC) were included in this analysis. After IMRT and at three months post-treatment the total number of voxels was 423 and 220, respectively. At three months post-treatment, a clinical assessment stratified patients into responders and non-responders. At six months post-treatment there was no residual disease in either group, as either the lesions completely responded to treatment (in 7 patients), or were resected with modified radical neck dissection (in 2 patients) [3].

**Fig 1:**
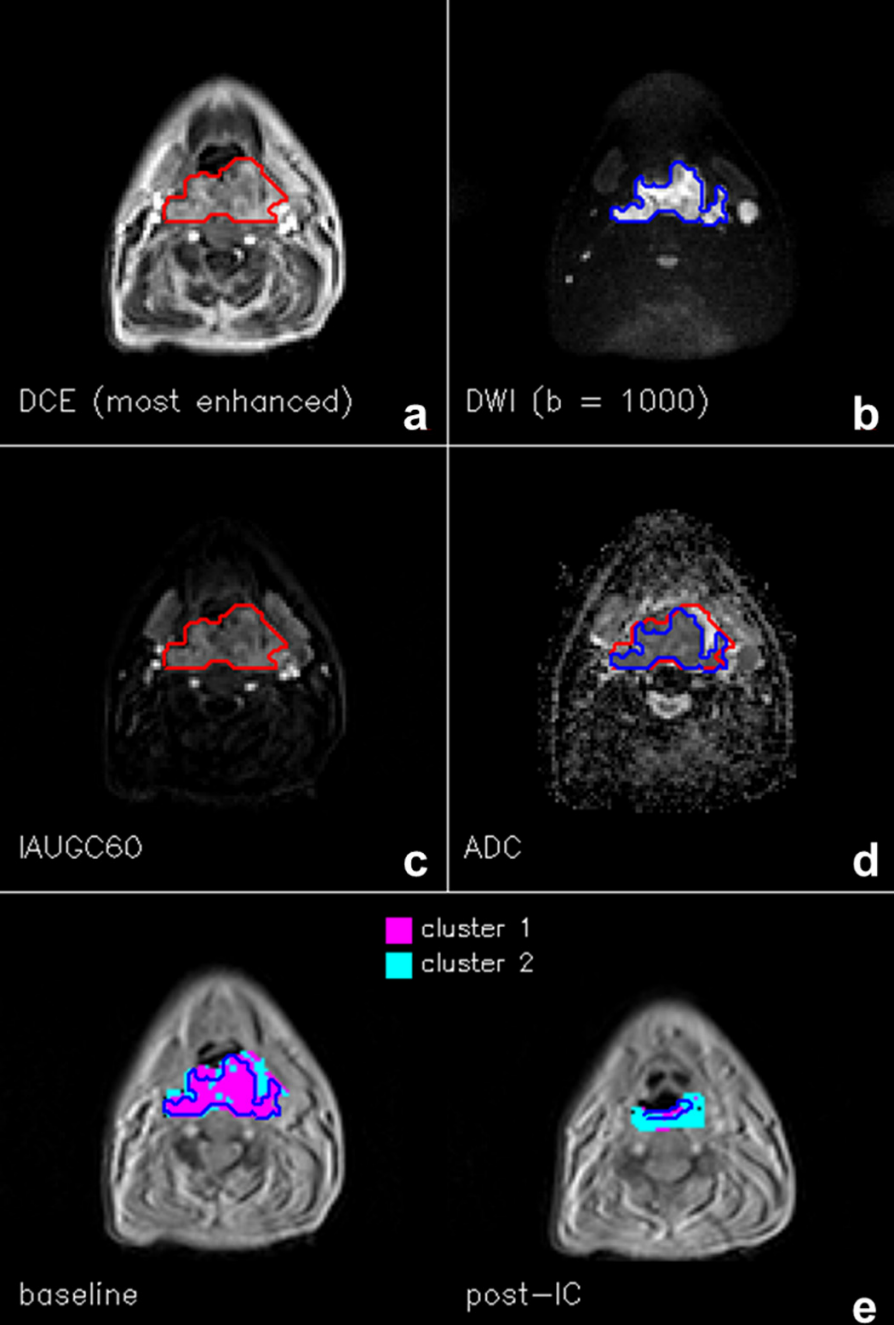
Slice with the largest tumour cross-section of a representative primary tumour. **(a)** ROI (red) contoured on the most enhanced frame of the DCE series, **(b)** ROI (blue) contoured on b = 1000 image of the DWI series, **(c)** IAUG60 map, **(d)** ADC map, with the two ROIs overlapped, and **(e)** cluster analysis map pre and post-induction chemotherapy (IC), in the simplest case of k = 2. The red ROI also encompasses the region where cluster analysis was performed; the blue ROIs represent the area of restricted diffusion independently contoured on DWI data, almost coincident with Cluster 1, which is characterized by both low ADC and high IAUGC60 values.

The ROIs contouring the area of enhancement on DCE data were employed in the analysis. For large PTs (an example in Fig 1) this area is larger than the core of restricted diffusion contoured on DWI data (a total of 970 and 703 voxels for all *central ROIs* at baseline, for DCE and DWI respectively), while for the LNs, which are generally encapsulated structures, there is minimal difference in size between DCE and DWI ROIs (a total of 475 and 490 voxels for all *central ROIs* at baseline, for DCE and DWI respectively).

The mutual information algorithm detected an in-plane shift in the phase-encoding direction of the DWI EPI images, due to offsets of the resonance frequency set by the scanner [7]. This occurred in 54% of the cases and was corrected by applying the computed integer voxel shift to the DWI images. Distortion in the MR images for this imaging protocol was previously assessed [16] and was minimal, leading to a reliable pairing between DCE and DWI voxels.

### Cluster Analysis

#### Principal component analysis

**Fig 2:**
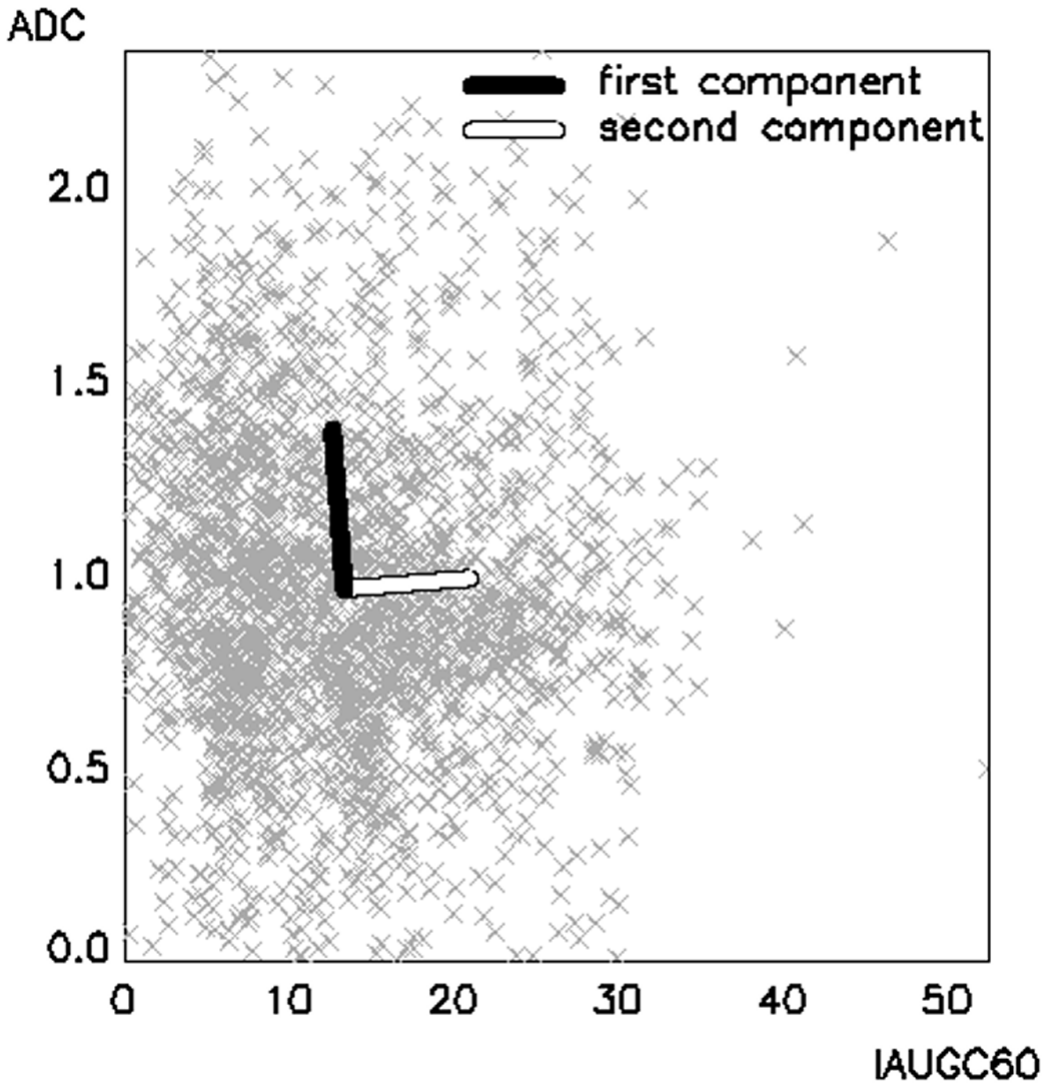
Principal component analysis. Principal component analysis in the bi-dimensional space formed by the two parameters IAUGC60 and ADC. The two vectors represent the two orthogonal components. The length of the two vectors is proportional to the weight of each component. IAUGC60 is expressed in units of mmol·s, ADC in units of ×10^−3^ mm^2^/s.

PCA analysis found that the two orthogonal components present in the data (Fig 2) are almost parallel to the original parameter axes (IAUGC60 and ADC), thus indicating that the two selected parameters are largely independent. The length of the two vectors in Fig 2 is proportional to the weight of each component. If it is assumed that the first component is almost coincident with ADC and the second with IAUGC60, it can be observed that their contribution is similar, with the first component accounting for 59.9% of the difference in the data.

#### Cluster validation

**Fig 3:**
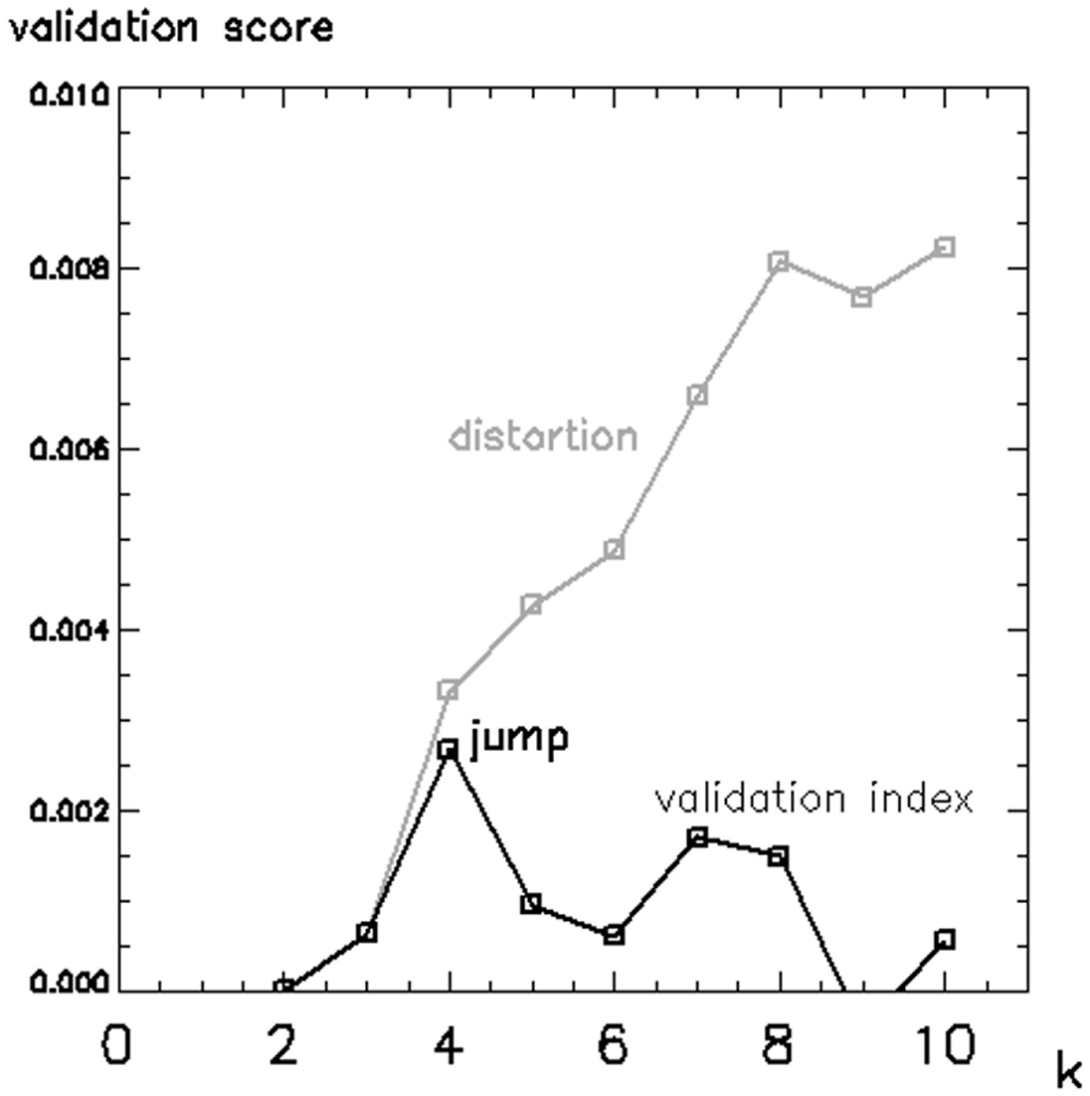
Cluster validation. Cluster validation curves for the range 2 ≤ k ≤ 10. The *distortion* curve (grey line) has a sharp rise in correspondence of the optimal number of clusters k = 4. The validation curve (black line) is produced by taking the difference between consecutive values of the *distortion* curve, and exhibits a “jump” in correspondence of k = 4.

Validation curves for 2 ≤ k ≤ 10 are presented in Fig 3, showing *distortion* as a function of the number of clusters (grey line). A sharp rise in *distortion* at k = 4 is associated with a sudden increase in performance of the clustering algorithm [15]. The validation curve (black line) offers an alternative visualization by plotting the difference between consecutive *distortion* values, and exhibits a “jump” at k = 4, which represents the optimal number of clusters.

#### Clustering algorithm

Considering the combination of PTs and LNs, Table 1 details the values of the cluster centres and the number of voxels in each cluster when k = 2, 3 or 4. The reported p-values indicate in which cluster the difference in the number of voxels pre and post-treatment reached significance; these clusters are characterized by high IAUGC60 values. Table 1 shows that with every choice of k it was possible to obtain these regions when partitioning the data, but with increasing k the algorithm was able to better separate clusters with a significant percentage reduction in voxel number following IC (83% and 81% with k = 4, p < 0.02) from clusters with a non-significant reduction (23% and 9% with k = 4, p > 0.75).

**Table 1:** Cluster analysis as a function of the number of clusters.

		Cluster 1		Cluster 2		Cluster 3		Cluster 4		Total	
**k = 2**	**Cluster centres**	IAUGC60 = 17.8, ADC = 0.73		IAUGC60 = 8.8, ADC = 1.21							
		**Baseline**	**Post-IC**	**Baseline**	**Post-IC**					**Baseline**	**Post-IC**
	**Voxel count**	828	285	607	459					1435	744
	**Reduction (%)**	66%		24%						48%	
	**p-value**	0.0074*		0.2005							
**k = 3**	**Cluster centres**	IAUGC60 = 22.5, ADC = 0.93		IAUGC60 = 9.6, ADC = 0.67		IAUGC60 = 9.1, ADC = 1.4					
		**Baseline**	**Post-IC**	**Baseline**	**Post-IC**	**Baseline**	**Post-IC**			**Baseline**	**Post-IC**
	**Voxel count**	574	95	500	374	361	275			1435	744
	**Reduction (%)**	83%		25%		24%				48%	
	**p-value**	0.0045*		0.9045		0.8181					
**k = 4**	**Cluster centres**	IAUGC60 = 22.2, ADC = 0.82		IAUGC60 = 19.7, ADC = 1.55		IAUGC60 = 9.9, ADC = 0.63		IAUGC60 = 7.2, ADC = 1.27			
		**Baseline**	**Post-IC**	**Baseline**	**Post-IC**	**Baseline**	**Post-IC**	**Baseline**	**Post-IC**	**Baseline**	**Post-IC**
	**Voxel count**	486	84	199	37	422	324	328	299	1435	744
	**Reduction (%)**	83%		81%		23%		9%		48%	
	**p-value**	0.0024*		0.0198*		0.7642		0.8572			

Results from cluster analysis (cluster centres and voxel counts) as a function of k (number of clusters), and percentage reduction after induction chemotherapy (IC) for the combination of primary tumours and lymph nodes. In total, 2179 voxels were considered in the analysis, from which 1435 were measured at baseline and 744 post-IC, with a global percentage reduction of 48%. Asterisks indicate significant two-tailed p-values (p < 0.05) from the Wilcoxon signed-ranks test, comparing the number of voxels pre and post-treatment. IAUGC60 (initial area under the gadolinium curve integrated over 60 s) is expressed in units of mmol·s, ADC (apparent diffusion coefficient) in units of ×10^−3^ mm^2^/s.

**Fig 4:**
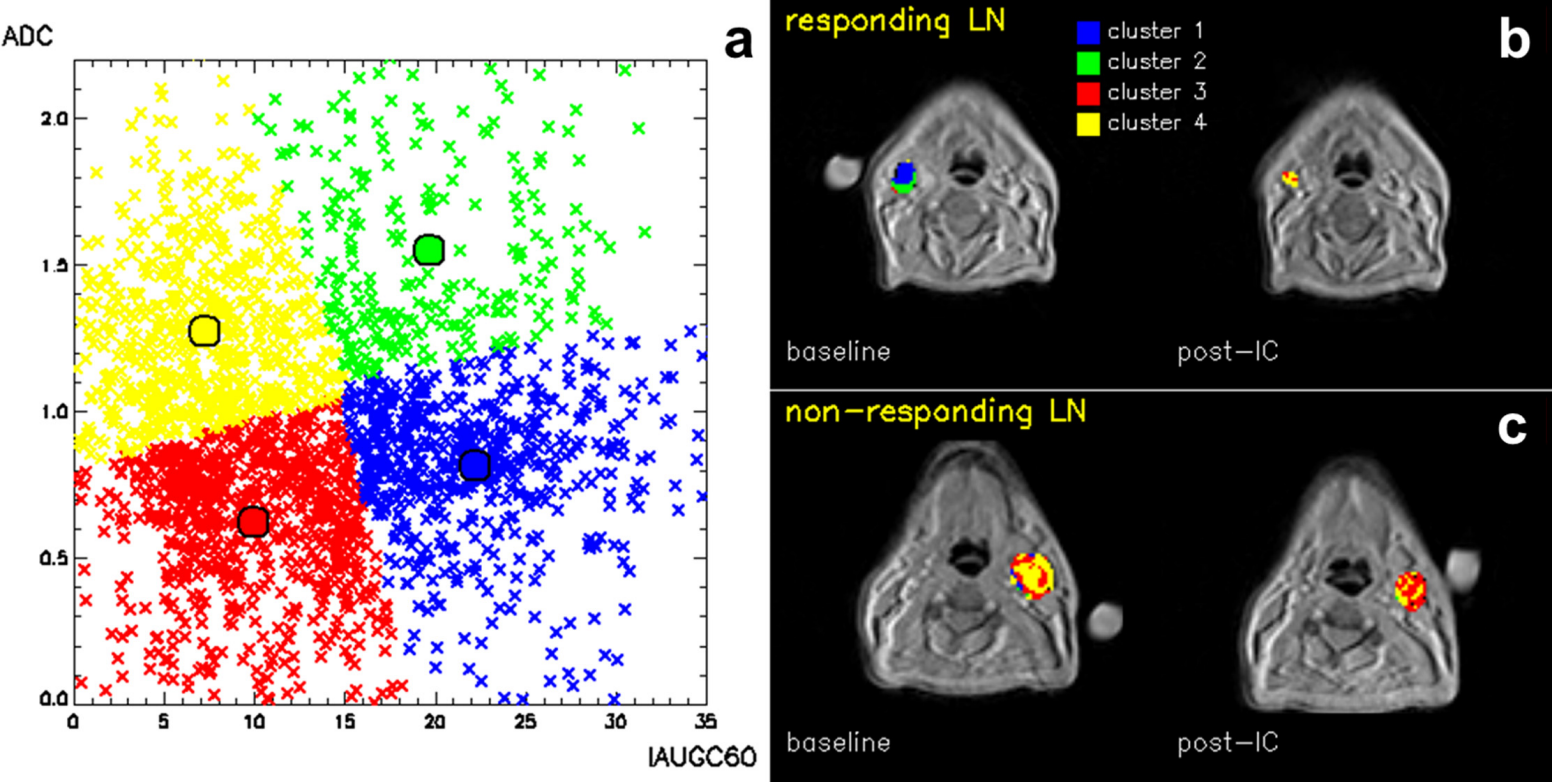
Cluster analysis. **(a)** Cumulative distribution of voxels (primary tumours + lymph nodes), partitioned with k = 4, in the bi-dimensional space formed by the two parameters IAUGC60 and ADC. IAUGC60 is expressed in units of mmol·s, ADC in units of ×10^−3^ mm^2^/s. **(b,c)** Corresponding cluster analysis maps of a responding (b) and a non-responding (c) lymph node, at baseline and post-induction chemotherapy (IC). Different colours indicate a difference in functionality at baseline.

Referring to the data analysed with k = 4, Fig 4 provides an example of the obtained colour maps, contrasting two LNs found to have different clinical outcomes assessed during long-term follow-up. The LN in Fig 4b, containing predominantly voxels belonging to Clusters 1 and 2 at baseline, responded to treatment, while the LN in Fig 4c, containing predominantly voxels belonging to Clusters 3 and 4, did not respond.

## Discussion

The proposed method robustly partitioned cumulative distributions of pre and post-treatment functional MRI data from SCCHN lesions into sub-sets of similar functional characteristics. In the cohort of patients analysed, this resulted in a description of functional heterogeneity.

The partitioning was based on two functional parameters derived from different MRI modalities: IAUGC60 and ADC. Wang et al considered two parameters derived from pharmacokinetic analysis of DCE data [9], but did not investigate a correlation between them. In our work PCA demonstrates that in this dataset IAUGC60 and ADC are independent parameters and have similar weight (Fig 2), thus providing distinct and complementary functional information. While the choice of IAUGC60 limits the specificity of the physiological information, it was preferred to more specific kinetic parameters like K^trans^ and v_e_, since it retains a relationship with these parameters but is unaffected by the accuracy of either the chosen contrast-agent uptake model or the arterial input function [4].

Our analysis was performed on data from both PTs and LNs. Limiting the analysis to the *central ROIs* reduces the unbalanced contribution of large lesions to the classification and minimizes inclusion of the voxels located at the lesion edges, where partial volume effects could be significant. However, one should verify that the slice with the largest tumour cross-section is representative of the tissue type content of the lesion (Appendix 1).

The evolution of the resulting sub-regions with IC treatment was assessed relative to the number of clusters (k = 2, 3 or 4); for all k, a statistically significant reduction in the number of voxels was found in post-IC data for clusters with high IAUGC60. This suggests that an enhanced delivery of IC in highly perfused areas might be the main determinant of response. A higher k allowed for a more complete description of heterogeneity, with optimal separation between clusters where the reduction in the voxel count post-IC is minimal and those where it is maximal.

Unsupervised clustering may be employed when, as in this case, there is no prior knowledge of the data. Nevertheless, determining the optimal number of clusters is essential to achieve meaningful results: a lower value could fail to differentiate some of the main structures in the data, while a higher value would simply increase the complexity of the partitioning without necessarily providing additional information. The cluster validation method that we adopted is non-parametric, makes limited assumptions, and has been proven effective in a wide range of problems for identifying underlying structures [15]. Table 1 shows that the separation between reducing and non-reducing clusters improves as k rises towards the optimal number of clusters (k = 4) mathematically derived with the jump method. This would appear to link mathematical computation with real functional differences, and informs the use of cluster validation to increase the reliability of unsupervised partitioning.

PCA and cluster validation can be employed as validation tools to investigate the composition of the data. In this case, both PCA (Fig 2) and the partitioning with the optimal number of clusters (Fig 4a) suggest that a binary classification of both parameters best separates the data, demonstrating consistency of results. Users should attempt to reproduce this consistency when applying this workflow to other datasets with different or higher number of clusters, higher data dimensions and possible internal correlations.

The analysed distribution of voxels included different tissue types before and after IC treatment. The adopted method performs an unsupervised partitioning of functional parameters in cumulative and longitudinal data, with the purpose of describing functional heterogeneity. Each pair of functional parameters describes tissue behaviour within a voxel, and as such, each voxel, rather than each lesion, contributes information to the global classification. However, the algorithm does not distinguish between pre and post-treatment data; it is therefore a remarkable result that this method independently separated voxels more likely to be affected by treatment.

This analysis was confined to baseline and post-IC data, because only small volumes of residual disease were present at subsequent time-points. Following IMRT treatment, response was dominated by volume reduction rather than by changes in functional parameters. In this situation, description of functional heterogeneity across a volume of few voxels is of limited value. Furthermore, in very small lesions both the weight and the reliability of the information extracted from the voxels are sensibly reduced. This type of analysis could have a potentially greater value at early stages of treatment response, where it could identify the sub-volumes whose functional parameter are more likely to be associated with treatment-resistant disease.

Each cluster represents a tissue class with a separate functional behaviour defined by the MRI parameters. Consideration of the cluster centre values, which summarize the functional parameters of each cluster, for the optimal k = 4 (Table 1) suggests the presence of regions of high vascular activity and regions that are more likely to be necrotic. Cluster 1 has increased perfusion (higher IAUGC60) and increased cellularity (lower ADC) and may represent a phenotype with rapid growth [23]. Alternatively, as a low ADC and high K^trans^ [24] at baseline predict treatment response [25], this phenotype may select tumours which are likely to respond to treatment, as our analysis seems to suggest. Cluster 2 separates areas with high perfusion and high ADC, suggesting the presence of oedema. The low perfusion associated with Clusters 3 and 4 could indicate necrotic tissue, whilst those voxels exhibiting a higher ADC in Cluster 4 might indicate the presence of liquid necrosis. However, histological validation is required in order to assign biological meaning to the clusters.

Functional heterogeneity within each lesion can be described by the presence of different clusters in each ROI. As an example, Fig 4 displays the cluster composition at baseline in two representative LNs: the non-responding LN is predominantly described by clusters with low IAUGC60 (Cluster 1 and 2), unlike the responding LN. The presence at baseline of those clusters which demonstrate low vascularity (low IAUGC60) would appear to be linked to treatment resistance. This suggests that the proposed analysis, limited to the initial response to IC, could have the potential to predict favourable and unfavourable prognostic groups.

The multi-parametric approach described in this paper differs from methodologies which consider single parameters separately and provide a description of their histogram distribution. In these cases, heterogeneity can be characterized by defining thresholds based on the analysis of the associated histogram. However, thresholds can not be determined independently and would necessitate evaluation of treatment response on a large number of cases. Furthermore, extension of threshold values to other datasets acquired with different MRI scanners and sequences is not straightforward, as thresholds rely on the numerical value of the parameter. Conversely, unsupervised cluster analysis, as presented here, produces sub-divisions as a function of distance in the multi-dimensional feature space without any prior knowledge. This type of analysis can therefore be transferred to other dataset, as it relies on the relationship between the parameters rather than their absolute numerical value.

The main aim of this paper is to present an approach to analyse longitudinal DCE and DWI data with the objective of characterizing tumour heterogeneity and response in SCCHN. The retrospective analysis performed on this pilot dataset is subject to a number of limitations. The low incidence of non-responding lesions precluded a statistically significant evaluation of the predictive value of our analysis. In addition, the absence of histological correlation limits the biological inferences which can be assigned to the clusters. This approach now warrants evaluation in a larger cohort of patients together with biological validation.

## Conclusion

We have presented a robust approach to partition cumulative distributions of functional parameters from SCCHN lesions, which employs unsupervised clustering, with no prior knowledge of the data, to separate voxels with distinct functional properties. In a distribution of pre and post-treatment MRI functional parameters, belonging to both PTs and LNs, the proposed methodology was able to separate clusters where the reduction in voxel number post treatment is maximal from those where it is minimal. Prior assessment of data composition and mathematical optimization of the number of clusters increased the reliability of the partitioning. This analysis demonstrated that independent functional parameters from different MRI modalities can be considered jointly to provide a multi-parametric characterization of functional heterogeneity within tumour volumes. By identifying those functional parameters more likely to characterize treatment-resistant disease, this method could have the potential to predict treatment outcome, to identify a biological target volume for treatment escalation or to stratify patients for alternative treatment modalities.

## Appendix 1

This section presents in detail the longitudinal analysis of a subset of the data containing pre and post-treatment PT *central ROIs*, partitioned with the minimum number of clusters (k = 2). Table 2 reports the number of voxels in each sub-region for each of the 5 PTs and in total, separating pre and post-treatment data. A global reduction in the number of voxels of 52% was observed, indicating response to treatment. Cluster 1 (Table 2, Fig 1e) is characterized by voxels with low ADC and high IAUGC60 values, associated with increased perfusion and cellularity. Fig 1e also demonstrates that this area is largely coincident with the area of restricted diffusion independently delineated on DWI data. The number of voxels assigned to this cluster reduced by 70% post-treatment. Voxels within this cluster were affected by treatment in greater proportion; these areas are both cellular and highly perfused, facilitating exposure of active tumour cells to induction chemotherapy. In contrast, Cluster 2 is characterized by low IAUGC60 and higher ADC values, and the voxel count reduced by only 7% post-treatment. While k = 2 might appear sufficient for partitioning the dataset containing only the PTs, the inclusion of the LNs required k = 4 to optimally characterize the higher data complexity introduced.

**Table 2:** (Appendix 1). Cluster analysis of the primary tumours.

		Cluster 1		Cluster 2		Total	
	Cluster centres	IAUGC60 = 17.3, ADC = 0.75		IAUGC60 = 8.1, ADC = 1.30			
Number of voxels		Baseline	Post-IC	Baseline	Post-IC	Baseline	Post-IC
	**Patient 1**	354	22	103	124	457	146
	**Patient 2**	187	100	23	11	210	111
	**Patient 3**	37	11	14	25	51	36
	**Patient 4**	38	14	5	13	43	27
	**Patient 5**	71	59	138	90	209	149
	**Total**	**687**	**206**	**283**	**263**	**970**	**469**
	**Reduction (%)**	70%		7%		52%	

Number of voxels, for each of the 5 primary tumours and in total, belonging to the two clusters determined by cluster analysis with k = 2. Pre and post-induction chemotherapy data are separated, and the total number of voxels in the ROIs is reported. IAUGC60 is expressed in units of mmol·s, ADC in units of ×10^−3^ mm^2^/s.

The 5 PT ROIs were also re-analysed comparing the use of the *central ROIs* only (1439 voxels) versus the use of the entire PT volumes (7067 voxels) for k = 2, 3 and 4, allowing investigation of the approach of including only the largest cross-section into the cumulative distribution. This is particularly relevant for PTs, which are often larger and asymmetrical structures. The two approaches provided similar sub-regions with any value of k, and cluster centre values no more than 9.1% apart (with an average difference of 4.3 ± 2.9% in the cases examined). This indicates that the analysis can be limited to the *central ROI* as the slice with the largest cross-section represents the tissue type content of the whole lesion, independently of the value of k adopted.
